# Forgery Detection and Value Identification of Euro Banknotes

**DOI:** 10.3390/s130202515

**Published:** 2013-02-18

**Authors:** Arcangelo Bruna, Giovanni Maria Farinella, Giuseppe Claudio Guarnera, Sebastiano Battiato

**Affiliations:** Image Processing Laboratory, University of Catania, Catania 95125, Italy; E-Mails: bruna@dmi.unict.it (A.B.); guarnera@dmi.unict.it (G.C.G.); battiato@dmi.unict.it (S.B.)

**Keywords:** banknote recognition, counterfeit detection, image forgery

## Abstract

This paper describes both hardware and software components to detect counterfeits of Euro banknotes. The proposed system is also able to recognize the banknote values. Differently than other state-of-the-art methods, the proposed approach makes use of banknote images acquired with a near infrared camera to perform recognition and authentication. This allows one to build a system that can effectively deal with real forgeries, which are usually not detectable with visible light. The hardware does not use any mechanical parts, so the overall system is low-cost. The proposed solution is reliable for ambient light and banknote positioning. Users should simply lean the banknote to be analyzed on a flat glass, and the system detects forgery, as well as recognizes the banknote value. The effectiveness of the proposed solution has been properly tested on a dataset composed by genuine and fake Euro banknotes provided by Italy's central bank.

## 1. Introduction and Motivations

The detection of counterfeit banknotes is one of the most important tasks in billing machines. Usually, it is performed by employing several image analysis techniques (transmittance analysis, reflectance analysis, *etc.*) with different light spectra (visible, infrared and ultraviolet). Unfortunately, these systems are very expensive and are employed only for ATM machines (*i.e.*, fully unsupervised by humans), where a high degree of reliability is required. In the last years, cheaper consumer systems for validation and classification of banknotes have been commercialized. They are usually based on motor actuators aimed at letting the banknote pass through light emitters and sensors in a dark area. This allows one to have controlled light and the correct positioning of the banknotes. On the other hand, such equipment is expensive, and the mechanical parts may wear out in a few years.

There are different motivations to produce low cost consumer validator machines for banknotes. Among others, there is the difficulty for blind and visually impaired people to recognize both the value and validity of banknotes. The World Health Organization (WHO) estimated that there were more than160 million visually impaired people in the world in 2002, which equates to more than 2.5% of the total population. Specifically, the study of the WHO reported that there were 124 million people with low vision and that 37 million were blind [1]. Although blind and visually impaired people can make a first screening on the value of the banknotes by taking into account the size of the different banknote values, there is no way for them to check for the validity and for forgeries in most cases. It should be noted that the validation of banknotes is a challenging task also for people without vision problems; indeed, under visible light, the counterfeit banknotes are usually identical to the valid ones. Since the encoding of new features to make the actual currencies “blind-friendly” has a high cost for the treasury departments of different countries [2], consumer cash validators can be very useful in bypassing this problem. Despite other works having addressed the problem of money recognition specifically for the visually impaired [3, 4], to the best of our knowledge, there are only a few works considering the problem of the validity of the money, as well as the problem of prototyping low cost hardware in this context.

In this paper, we present both hardware and software components useful in detecting counterfeits of Euro banknotes and in recognizing their currency value. This is obtained by exploiting a low-cost system without motor actuators or other mechanical parts. A prototype has been built and tested on a dataset composed by genuine and tampered Euro banknotes provided by the Bank of Italy [5]. The system is composed mainly by an infrared camera, a microprocessor and a software component implementing smart algorithms for counterfeit detection and value recognition. A glass is placed in the focal plane of the camera in order to acquire sharp banknote images. The acquired images are then processed through the designed algorithms. The user should just lean a banknote on the glass, and the system provides the information on the validity and the related value.

The remainder of the paper is organized as follows: Section 2 reviews the state of the art approaches in the field. Section 3 summarizes the proposed algorithms for counterfeit detection and currency value recognition of Euro banknotes. In Section 4, the designed hardware prototype is described, whereas experimental results are presented in Section 5. Finally, conclusions and future work are given in Section 6.

## 2. State-of-the-Art

It is well known that, in order to avoid forgeries, several security systems are typically encoded into banknotes (in different ways for each currency) [6]. This fact has induced researchers to develop different counterfeit detection algorithms, taking into account the different currencies. Despite different algorithms having been presented in the literature for embedded device systems [7, 8], just a few researchers proposed a full system for both recognition and validation of Euro banknotes considering a constrained domain.

The authors of [3] make use of light transmittance and pattern recognition techniques to classify the value of banknotes. The method requires the banknote to pass through light emitters (LED) and receivers (photo transistors) placed on the opposite side. Hence, the system requires motor actuators and other mechanical parts. Moreover, the method can be simply deceived by counterfeit banknotes (not handled by the authors).

In [9], an algorithm for Korean Won bill classification is described. It makes use of images acquired in the visible light spectra. The recognition is performed by extracting features in the wavelet domain. Banknote images are first processed with a Sobel filter, and then, the wavelet feature extraction is performed. For classification purposes, the canonical analysis projection is considered in order to reduce the feature space dimensionality, obtaining a more discriminative space. The K-nearest neighbor algorithm is employed for final banknote recognition. Also, in this case, the problem of detecting counterfeit banknotes is not considered. Moreover, since the classification approach is memory-based, the algorithm cannot be used, as it is in consumers' cash machines, where the constraint of having low-cost hardware imposes strong limitations in memory usage.

The problem of Sterling banknote validation is addressed in [10]. The validation is obtained through a cascade of segmentation and classification procedures. The images of banknotes are first segmented in different regions, and then, the classification results on the different regions are combined to perform the final banknote authentication. A genetic algorithm has been used to distinguish valid and counterfeit banknotes. Since the approach considers Sterling currency, the applicability is not straightforward in the context of Euro banknotes, because this currency encodes different strategies to avoid forgeries, and hence, different assumptions on features and their positioning should be done.

Neural network and genetic algorithms have been exploited in [11, 12] to address the problem of banknote recognition. The paper focuses mainly on how to optimize the masks exploited by a neural network to perform value recognition. Genetic-based mask optimization is proposed by the authors to effectively generate the characteristic values of the input banknote image. The banknote values recognition task has been experimentally demonstrated on different world-wide currencies that were present at that time (e.g., the old Italian Lira, German Marks, Spanish Pesetas, *etc.*). Unfortunately, this approach does not take into account the problem of banknote counterfeit detection, which is the main problem addressed in this paper.

In [4], a framework that employs Speeded Up Robust Features (SURF) for US banknote recognition has been proposed. The system makes use of pre-computed SURF features collected on 14 images of the front and back sides of seven categories of bills ($1, $2, $5, $10, $20, $50 and $100) as ground truth. Given a new US banknote image to be recognized, SURF features are extracted and then matched with respect to the ground truth dataset. The numbers of matched features and the spatial relationship between them are used for the value recognition. A drawback of this approach is that it could fail with simple counterfeit banknotes (e.g., a photocopy) because forgeries can usually be captured employing analysis in a light domain different from the visible one (e.g., infrared spectrum).

The Taiwanese currency has been considered in [13]. The method proposed to detect counterfeit banknotes is based on multiple-kernel support vector machines. Banknotes are firstly divided into partitions, and the luminance histograms of the partitions are taken as the input of the system. Each partition is associated with its own kernels. A linear combination is adopted to exploit multiple kernels for classification purposes. Despite the method showing good performances, the overall approach is not suitable for embedded constrained domains, where the hardware to be used has very limited resources. Moreover, since the approach works in the visible spectra, forgeries cannot be addressed by this system.

The color domain has been considered in [14] for US banknote validation. In this case, each banknote part has been represented as a color histogram after a subdivision of the input image in regions. The fuzzy Hamming distance has been employed during classification. Despite the results on simulated counterfeit banknotes showing good performances, in the case of Euro banknotes, the real forgerie scan not be distinguished just by using features based on color.

In [15], the authors proposed an Intelligent Banknote Identification System (IBIS) based on the neural network technique. The system is designed for Turkish Lira and Cyprus Pound identification. Also, in this case, the problem of tampering has not been taken into account.

The Euro banknotes have been considered in [16]. The recognition system works with images acquired in the visible light spectra and uses two types of neural networks. The authors used a three-layered perceptron for recognition and a RBF (Radial Basis Function) network for the rejection of invalid data. The approach has been tested only on simulated data for the rejection case. It should be noted that most of the tampered banknotes looks like the genuine one, and a robust cash validator cannot be obtained using just the visible light spectra.

**Figure 1.**
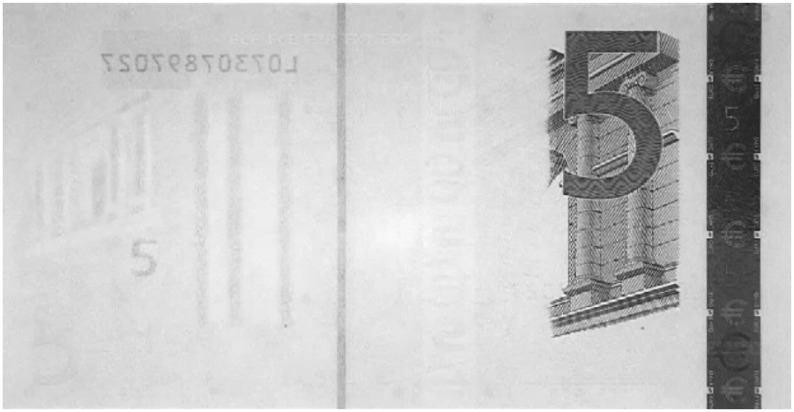
Among the various security features, the one encoded into the infrared light spectra is incorporated into all euro banknotes to protect them against counterfeiting [6]. Here is reported an example of five Euro banknotes under infrared light, as it is shown on the official website of the European Bank [6]. Only the right side of the main image and the silvery stripe are visible.

**Figure 2.**
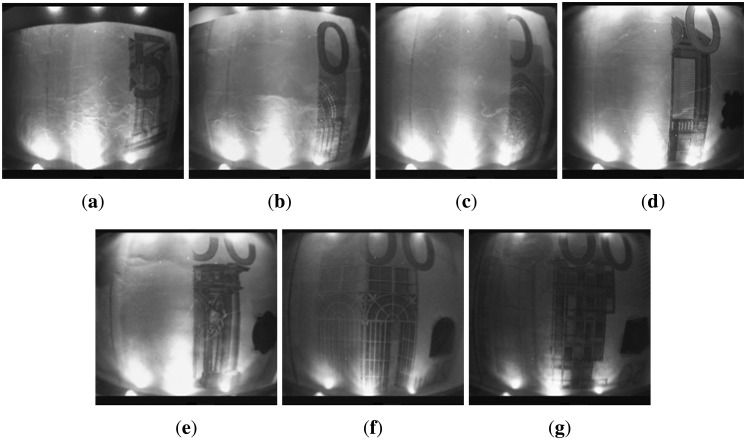
Examples of banknote values acquired by the infrared (IR) camera of the proposed system. A simple visual comparison between the above banknotes and the one in Figure 1 reveals the different problems (e.g., distortion, illumination variation, misalignment) that should be taken into account in building a low cost counterfeit detection system. (**a**) 5 Euro;(**b**) 10 Euro; (**c**) 20 Euro; (**d**) 50 Euro; (**e**) 100 Euro; (**f**) 200 Euro; (**g**) 500 Euro.

Despite different approaches having been presented in the literature, most of them do not take into account the Euro banknotes or the authentication task. Moreover, some approaches have been tested only on simulated data. All the techniques above could be adapted to other currencies (e.g., the Euro) for the recognition task, but their adaptation to counterfeit detection is not straightforward, because, under visible light, the counterfeit banknotes usually look identical to the valid ones. Hence, a system designed for a specific currency (e.g., the Euro) cannot be employed, as it is for other currencies (e.g., Sterling, Turkish Lira); each currency has its own specific features to be exploited for counterfeit detection. Typically, those features strongly differ from one currency to another. In the Euro banknotes, there are several features that can be used to detect forgeries: the sheet, the watermarks, the particular inks with different behavior in visible, infrared and ultraviolet lights, *etc.* (see [6] for a full overview). Different than other approaches, in the proposed system, the infrared (IR) technology has been exploited, since it can provide good and robust features for counterfeit detection of Euro banknotes. Examples of Euro banknotes acquired with the IR light are shown in Figures 1 and 2. The two different inks present on the Euro banknotes (one reflective to IR and one absorbing IR) allow one to obtain powerful features for the involved tasks (*i.e.*, counterfeit detection and value recognition).

## 3. Validation and Recognition

**Figure 3.**
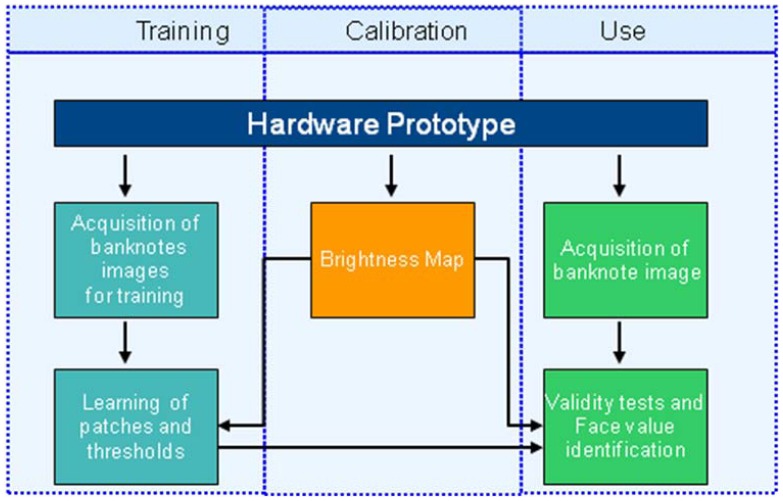
Overall schema of the proposed approach.

The overall scheme of the developed algorithm for infrared-based counterfeit detection and currency value recognition is shown in Figure 3. The overall pipeline consists of three main blocks: calibration, training and use module. To build a low cost cash validator, we need to consider different constraints (*i.e.*, very low memory usage, a low cost acquisition camera, *etc.*), which make the recognition and counterfeit detection tasks more challenging. The proposed software solution has been designed, taking also into account low cost hardware with limited resources, detailed in Section 4.

### 3.1. Calibration

Since, in our setup, the LED illumination is not spatially uniform (see Figure 2), and taking into account that illumination of the proscenium could change (We assembled two instances of the proposed cash machine to understand and test the variability of both settings and results), a calibration phase is required to remove variability. The light variability can be considered a fixed pattern noise [17], since it is constant along time. Specifically, in this phase, a brightness map is computed, and a compressed version of it is stored inside the flash memory (*i.e.*, in non-volatile memory). To obtain the brightness map, an image of a white sheet of paper is acquired under idealized lighting conditions (*i.e.*, in a darkroom, with no external source of infrared light). Since the captured frame is very noisy, a multi-frame acquisition is performed, and the map is obtained as the average of such frames. This process also allows noise reduction (usually zero mean Gaussian noise [18]). The map is then used during the processing modules (training and use) to normalize the input banknote images as follows:

(1) $$\text{NormalisedImage}(i,j)=\frac{\text{InputImage}(i,j)}{\text{Brightness Map}(i,j)}$$

### 3.2. Training

The Training block is used to learn the optimal features of the banknotes (*i.e.*, template patches), which will be used to determine the validity and the value of the banknotes. The parameters are learned using a large data set of both genuine and counterfeit banknotes, which come in several face values.

The main problem in this stage is the availability of training data. Indeed, it is not simple to obtain real counterfeit banknotes, especially when infrared light is used as the feature extraction domain. Hence, most of the work in the literature uses the visible light domain for feature extraction and is tested on simulated counterfeit banknotes. However, simulated forgeries (e.g., photocopies) are the most simple to detect and usually can be handled directly by humans without further analysis by a cash machine validator. Here, we consider the task of dealing with counterfeit banknotes that look identical to the genuine one under human inspection (*i.e.*, under visible light). To this aim, in our tests, we have used both genuine and real counterfeit banknotes collected, only for testing purposes, at the Bank of Italy [5].A training dataset of 1, 000 images has been acquired, taking into account the typical contexts of the usage of the apparatus, with very different lighting conditions (e.g., neon, sunlight, incandescent and fluorescent lamps, *etc.*) and a high degree of misalignment with respect to the proscenium (the system must be robust to slightly translated and/or rotated banknotes). Genuine Euro banknotes are printed such that only specific visual features are visible under infrared lighting. The two different inks present on the Euro banknotes (one reflective to IR and one absorbing IR) allow one to obtain powerful features for the involved tasks (*i.e.*, counterfeit detection and value recognition). In particular, under infrared light, banknotes must show darker areas in different zones depending on their value (All the properties to detect counterfeits of the Euro banknotes (e.g., infrared light) are available at the official website of the European Bank [6]). Those areas always show characteristic patterns useful for the recognition task. In Figures 1 and 2, some examples of the different banknotes values acquired by the IR camera of the proposed system are reported. Note that a counterfeit banknote (e.g., like a photocopy) could show the same patterns from a genuine one under visible light, but under IR light, the forgery is typically revealed (e.g., a photocopy does not show patterns under IR light). The overlap, of both dark and bright are as, among all considered banknote values under infrared light, can be exploited for the validity check. Despite the simplicity of the features, these are the only ones that the bank controls and accepts in order to assess the validity of the Euro banknotes. Our experiments demonstrated that such features are strong enough to discriminate between genuine and counterfeit banknotes. The aim of the learning stage is, hence, to find the best areas in order to check the banknote validity by taking into account the training dataset composed by genuine and fake Euro banknotes. We would like to remind the reader that the hardware constraints (see Section 4) do not allow the exploitation of most state-of-the-art methods in computer vision and pattern recognition for feature extraction (e.g., SIFT) and recognition (e.g., SVM).

Given the set of all genuine banknotes, *G* = {*G*_1_, *G*_2_, …, *G_n_*}, the training block is devoted to searching for the largest common dark area, *F_g_*, and the largest common bright area, *T_g_*, of genuine banknotes.

Let *F_g, i_* = *IR_Signal_*(*G_i_*) be the infrared highlighted dark area of each *G_i_*, then we define:

(2) $$F_{g}=\underset{i}{\cap }F_{g,i}$$

Let *T_g, i_* = *G_i_\ F_g, i_* be the unresponsive bright area of each *G_i_*, then we define:

(3) $$T_{g}=\underset{i}{\cap }T_{g,i}$$

The regions *T_g_* and *F_g_* need to be refined in order to be robust to counterfeit banknotes, which in some cases might show a slightly similar infrared response (e.g., 5 and 20 Euro). Given the set*C* = {*C*_1_, C_2_, …, *C_n_*} of all counterfeit banknotes of our dataset, we define:

(4) $$F_{c,j}=\text{IRSignal}(C_{j})$$

and

(5) $$T_{c,j}=C_{j}\backslash F_{c,j}$$

Let C* be the set of all *C_j_* such that:

(6) $$(F_{c,j}\cap F_{g}\neq ⊘)\wedge (T_{c,j}\cap T_{g}\neq ⊘$$

By considering C* and previous equations, we can define the regions *T* and *F* to be checked as follows:

(7) $$T=T_{g}\backslash \underset{j}{\cup }T_{c,j}$$

and

(8) $$F=F_{g}\backslash \underset{j}{\cup }F_{c,j}.$$

**Figure 4.**
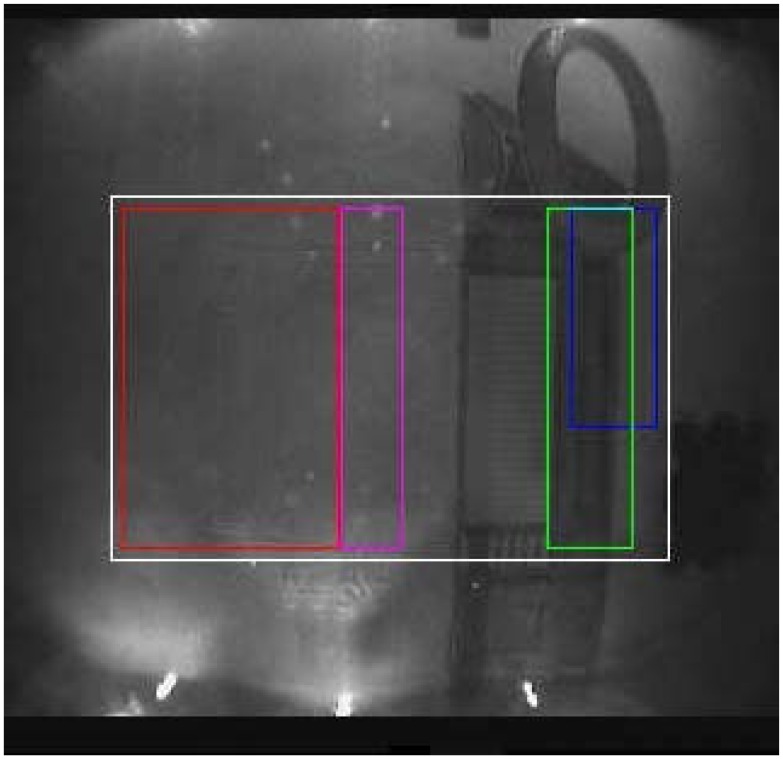
A banknote of 50 Euro as acquired by the IR camera of the proposed system, and the set of regions used to test its genuineness. White: overall considered region of interest. Blue: the threshold to binarize the image is calculated in this area. Green: this area must bedark under infrared light. Red: when acquired under infrared light, this area must be bright and without noticeable patterns. Magenta: additional area used to check the genuineness of some denominations.

For each banknote, *B_k_*, in *G* ∪ *C* and for each possible threshold, *s* ϵ [0, 255], we computed the percentage *percB_k, s_* of pixels above *s* in the region *T_k_* and the percentage *percD_k, s_* of pixels below *s* in the region *F_k_*. Using those values, we computed the optimum percB* and percD* thresholds to discriminate between genuine and counterfeit classes. Figure 4 reports the region *T* (*i.e.*, magenta/red bounding box) and *F* (*i.e.*, green bounding box) to be checked for the banknote analysis.

**Figure 5.**
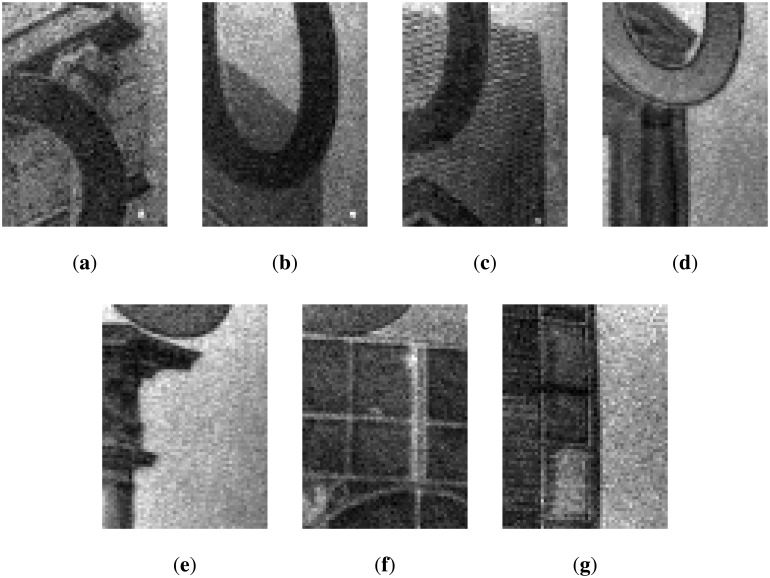
Learned patches related to the different banknote values, (**a**) 5 Euro; (**b**) 10 Euro;(**c**) 20 Euro; (**d**) 50 Euro; (**e**) 100 Euro; (**f**) 200 Euro; (**g**) 500 Euro.

To classify the banknote value, during the training phase, we learn the most discriminant patterns, *P_i_*, for each possible banknote value, *D_i_* ϵ *D* = {5, 10, 20, 50, 100, 200, 500}. Specifically, we learn patterns such that the intra-class distance is minimized, whereas the inter-class distance is maximized. As in the case of validity, this process is very tricky for some banknote values that share similar patterns(e.g., 5 and 20 Euro). The final output is a set of patches (with the corresponding position) related to the different banknote values (see Figure 5). To take into account slightly translated and/or rotated images, the search area of each patch has the same shape and center coordinates of the patch itself, but it is wider. The search area is approximately 2.5-times the corresponding patch area.

Once the training phase is performed, the selected banknote features (*i.e.*, the learned patches, locations and thresholds) are stored in the flash memory of the device and used to infer the validity and value of the input banknote images (see Figure 3).

### 3.3. Banknote Authentication and Recognition

Once the image has been corrected from the non-uniform LED illumination problems (see Section 3.1), a suitable threshold needs to be found according to the actual input data. In our experiments, we have noticed that the average gray value (indicated as *MeanRef*) of the blue region shown in Figure 4 can be robustly used for this purpose. To check the genuineness, we compute the percentage of pixels inside the green region with a gray value below *MeanRef* (*i.e., percLT*), together with the percentage of pixels inside the magenta and red regions with a gray value above *MeanRef* (*i.e., percGT*). The acquired banknotes are hence classified as genuine by using the following formula:

(9) (percLT>percD*)∧(percGT>percB*)

where percD* and percB* are learned, as described in the previous section.

To identify the face value, the system makes use of patches learned during the training stage (*i.e.*, the templates related to the different banknote values reported in Figure 5) and the corresponding search area coordinates. Search areas are wider than the patches in order to be robust to small misalignments. Each template patch is placed at the center of its search area, and a correlation measure between the pattern itself and the corresponding pixels on the search area is used for comparison. The procedure then searches if a translation around the neighborhood of the current position could increase the correlation. This is performed by moving the pattern position. This step is then repeated until the correlation reaches a local maximum. The pattern with the highest correlation determines the final value to be assigned to the banknote given as input. Information about genuineness and face value are then sent to the display, in order to inform the user about the results of the banknote analysis (Audio signals can be used at this stage to perform transduction of the information on validity and value of the Euro banknotes in order to extend the accessibility to visually impaired and blind people). It should be noted that the proposed software has been designed with the aim to work with low cost hardware and, hence, with low computational resources (*i.e.*, 32 k byte of internal RAM and 256 k byte of non-volatile memory to store the binary code and all the templates). The proposed solution needs 1.88 k byte to store the calibration map, 28.2 k byte for the templates to be used for value recognition, and 114 k byte for software instructions. Using the architecture described in the Section 4, the overall computational speed is about 1 second for validity and 2 seconds for both validity and value recognition.

## 4. HW Prototype

**Figure 6.**
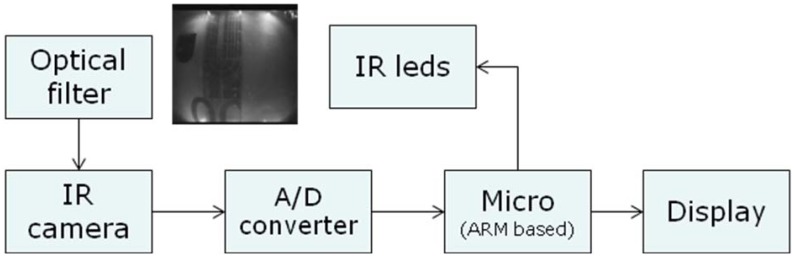
The schema of the hardware prototype.

A hardware prototype has been designed and developed to demonstrate the effectiveness of the proposed framework. The overall system is summarized in the block diagram reported in Figure 6.

The Infrared LEDs (IR LEDs block) illuminate the scene. This block is composed by six LEDs placed around the proscenium (*i.e.*, the area where the banknote is placed). The illumination is not uniform in the real system. Hence, a calibration is needed to take into account the non-uniformity and the performance decay during the system life (see Section 3.1). An optical filter is inserted to avoid an external light source from influencing the image acquisition system. The filter is placed on top of the infrared camera (IR camera block) that acquires the image. The camera is a common low cost camera with CCTV output. Since the output is analog, an analog to digital converter (A/D converter block) is used to obtain a digital standardized format (*i.e., CCIR* – 656). The digital image is then processed by the microprocessor. In the prototype, we have used a main board *AT MEL AT91SAM9XE*256 with a*ARM926EJ* — *S*™ processor at 200 MHz and 256 KB internal high-speed flash memory, which is used to store the program instructions. The board includes also an Image Sensor Interface (ISI) port able to capture video sequences compliant with the standard ITU-R BT 601/656. The software to be run in the proposed hardware is composed by the control logic for the entire subsystems (e.g., IR led, IR camera settings, Display, *etc.*) and the related algorithms (for both validation and classification) described in Section 3. The prototype has been also equipped with 4 M byte of external SRAM memory, since the microprocessor contains only 32 Kbyte of internal SRAM.

**Figure 7.**
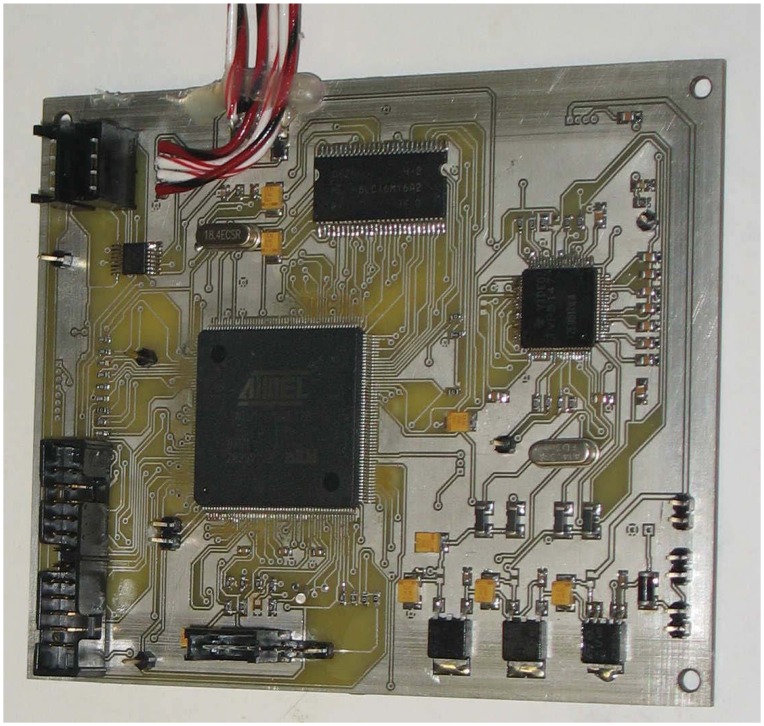
The main board containing the microprocessor in the center, the A/D converter on the right side, the external SRAM memory in the upper part and the power supply in the lower/right side.

**Figure 8.**
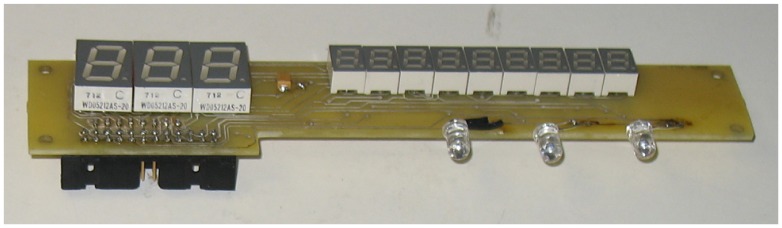
The display board containing eight-segment displays. Also, three IR LEDs are visible.

**Figure 9.**
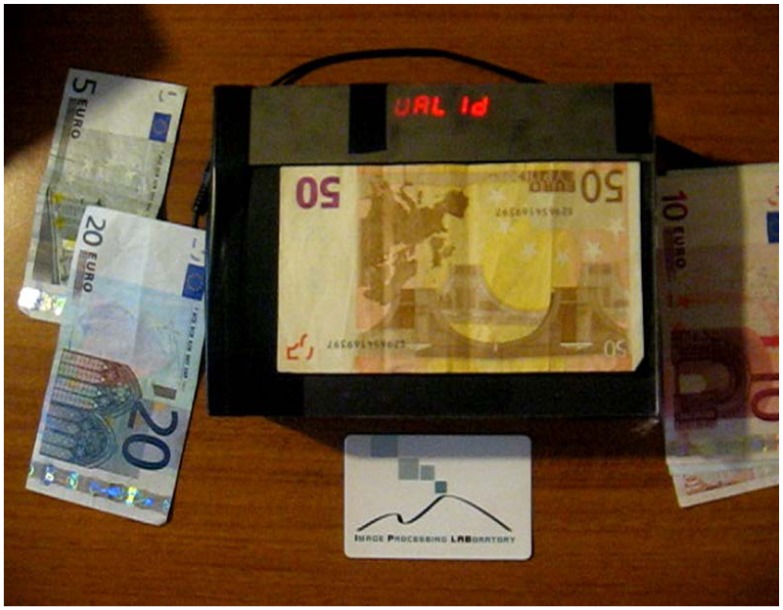
The final prototype of our system.

In Figure 7, the main board and the other hardware components composing the prototype are shown. In the center, there is the ATMEL microprocessor; on the right side, there is the A/D converter; in the upper part, there is the external SRAM memory; in the lower/right side, there is the power supply. The display used to show the output of validation and recognition of banknotes is composed by several eight-segment displays (Figure 8): three are used to show the banknote size, while eight are used to show the validity and other service messages. The final prototype in operation is shown in Figure 9. The overall hardware has an estimated cost of 30$ when produced in a large scale.

## 5. Experimental Results

To evaluate the performance of the proposed technique, we used our prototype to acquire 2, 750 images to be used as a training and test set. Tests have been repeated five times. The reported results are obtained, averaging among the different runs. At each run, a training dataset of 1, 000 images has been considered, taking into account the typical contexts of usage of the apparatus, with very different lighting conditions(e.g., neon, sunlight, incandescent and fluorescent lamps, *etc.*) and a high degree of misalignment with respect to the proscenium (the system must be robust to slightly translated and or rotated banknotes). A test set of 1, 750 banknote images, acquired with the same criteria used for the training dataset (*i.e.*, both counterfeit and genuine banknotes have been acquired under several environmental lighting conditions, with different illuminants and brightness) has been used for testing purposes. The statistical properties of the features are reported in Table 1. Taking into account the learning stage described in Section 3.2, the values of percD* and percB* used in our experiments are, respectively, 0.52 and 0.85.

In both cases, *i.e.*, training and experimental phases, the banknote samples (genuine and counterfeit) have been provided by the Bank of Italy [5]. Unfortunately, a comparison of the counterfeit detection performance between the proposed method and the ones reviewed in Section 2 is not possible (or fair), since the reviewed approaches in the literature do not consider the problem of validation and/or have been designed to consider other currencies or different light spectra to extract the features.

Acquired counterfeit banknotes include also specimens carefully calibrated to mislead digital counterfeit detectors. The specimens are used by the bank to test the new cash validators before they can obtain a certification for commercialization. A cash machine should be able to recognize both values and counterfeit banknotes proposed by the bank to obtain certification. To deal with special fake banknotes provided by the Bank of Italy, additional procedures have been included in our software to deal with extreme, but real, forgeries on banknotes (per a NDA with the company supporting this activity, we cannot reveal how those forgeries appear in the infrared spectrum). Despite the overhead needed to compute some specific features in order to deal with the learned patterns on specimens, the overall processing time matches one of the base algorithms presented in Section 3. Indeed, the final counterfeit software includes just a few more lines of code to perform some computations on very small areas of the acquired images. Table 2 reports the results of the tests for the validity assessment, whereas Table 3shows the results of the banknote value classification.

Most of the false negatives are observed for images acquired under direct sunlight illumination and for very worn banknotes. Wear and tear can be identified as the main cause of misclassified samples for5 and 10 Euro banknotes, since those banknotes are widely used in everyday life.

## 6. Conclusions and Future Work

In this paper, we have proposed an effective system composed by both hardware and software modules to detect counterfeits of Euro banknotes. Conversely, to the state-of-the-art algorithms, the proposed solution makes use of infrared imaging and low-cost hardware. The proposed system allows recognizing not only the value, but also forgeries. The described algorithms are robust to changes in environmental lighting, in terms of illuminant type and incident intensity. Thanks to a training phase, it is also robust to non-uniformity of the infrared light. The experiments performed on real genuine and fake banknotes provided by the Bank of Italy demonstrated good performances in both validity and value recognition. Future work will be devoted to increase the recognition accuracy and to reduce the computational time. Different feature spaces and classification algorithms suitable to work in constrained domains will be tested [8]. Moreover, extension of the current solution to other currency (e.g., US Dollars, UK Pounds, etc.) will also be considered. Finally, the capability of the prototype will be augmented in order to allow audio signals as output and, hence, giving the possibility to convert both validity and values information for visually impaired and blind people.

## Figures and Tables

**Table 1.** Statistical properties of the features.

		**Min**	**Max**	**Avg**	**Std**
**percLT**	**Genuine**	0.5006	0.6757	0.5864	0.0451
	**Counterfeit**	0.1802	0.5437	0.4412	0.1109
**percGT**	**Genuine**	0.8741	0.9972	0.9722	0.0431
	**Counterfeit**	0.5705	0.8322	0.6436	0.0975

**Table 2.** Genuine/counterfeit classification.

**True Positive(Counterfeit banknote correctly classified)**	**False Positive(Genuine banknote incorrectly classified)**
100%	4.3%
**False Negative(Counterfeit banknote incorrectly classified)**	**True Negative(Genuine banknotes correctly classified)**
0%	95.7%

**Table 3.** Banknote value classification.

	**5E**	**10E**	**20E**	**50E**	**100E**	**200E**	**500E**	**Counterfeit**
5E	88%	0%	0%	0%	0%	0%	0%	12%
10E	1%	91%	0%	0%	0%	0%	0%	8%
20E	0%	0%	98%	0%	0%	0%	0%	2%
50E	0%	0%	0%	99%	0%	0%	0%	1%
100E	0%	0%	0%	0%	93%	0%	0%	7%
200E	0%	0%	0%	0%	0%	98%	1%	1%
500E	0%	0%	0%	0%	0%	0%	95%	5%
